# Regulatory Pathways That Facilitated Timely Registration of a New Group A Meningococcal Conjugate Vaccine for Africa's Meningitis Belt Countries

**DOI:** 10.1093/cid/civ491

**Published:** 2015-11-09

**Authors:** Nora Dellepiane, Bartholomew Dicky Akanmori, Sunil Gairola, Suresh S. Jadhav, Cathy Parker, Carmen Rodriguez, Swati Srivastava

**Affiliations:** 1 Department of Essential Medicines and Health Products, World HealthOrganization, Geneva, Switzerland; 2 Immunization, Vaccines and Emergencies, Regional Office for Africa, World Health Organization, Brazzaville, Republic of the Congo; 3 Serum Institute of India, Ltd, Pune; 4 Biologics and Genetic Therapies Directorate, Health Canada, Ottawa; 5 Central Drugs Standard Control Organization, Drug Controller General of India, New Delhi

**Keywords:** prequalification, national regulatory authorities, fast-track procedure, market authorization, low- and middle-income countries

## Abstract

***Background.*** Through its normative and public health leadership roles, the World Health Organization (WHO) plays a key role in the availability of vaccine products in low-and middle-income countries. The recent introduction of a new group A meningococcal conjugate vaccine, PsA-TT (MenAfriVac), in Africa exemplifies this process. WHO requires that any new vaccine to be introduced in countries for public health reasons and supplied through United Nations centralized mechanisms be licensed by the national regulatory agency (NRA) in the producing country, then prequalified and given a marketing authorization in the user countries.

***Methods.*** PsA-TT was manufactured by the Serum Institute of India, Ltd (SIIL), which submitted a license application in April 2009 to the Drug Controller General of India (DCGI), the Indian NRA responsible for licensing vaccines. WHO encouraged the DCGI to establish a collaboration with Health Canada's Centre for Vaccine Evaluation for the review. Through this collaborative effort, registration was facilitated and in December 2009 an export license was granted to SIIL, which subsequently submitted an application for WHO prequalification.

***Results.*** Given the importance of the vaccine, WHO “fast tracked” the prequalification review, and after a detailed review and site visit, WHO prequalification was granted to PsA-TT in June 2010. Country use of the new vaccine could not occur until the vaccine was a registered product in each country seeking its use. WHO facilitated country reviews by conducting regulatory training exercises (in French and English) for country NRA staff, which used the PsA-TT registration as a case study.

***Conclusions.*** PsA-TT was gradually registered in African countries as vaccine introduction proceeded. The regulatory pathway for this new group A meningococcal conjugate vaccine proved to be a useful training opportunity both in India and Africa, because the availability of the vaccine was a high African public health priority, as well as for WHO as a case study to facilitate registration of vaccines based on reliance on other regulatory bodies.

All vaccines under development follow a predefined pathway that begins with nonclinical testing (in suitable animal models and in vitro testing), followed by a stepwise evaluation in human clinical trials to demonstrate safety and efficacy. After clinical results are carefully reviewed and deemed satisfactory, a candidate vaccine can be registered and granted marketing authorization. Vaccine clinical trials are carefully designed human studies aimed at systematically assessing safety and efficacy. Regulatory oversight of clinical trials is critical to ensure that vaccines under development meet the required quality, safety, and efficacy standards [1, 2]. Data from the trials are submitted to national regulatory agencies (NRAs) for registration of the vaccine before the vaccine can be used.

International guidelines define the criteria and review processes that are used to register vaccines. In general, clinical trial applications for new vaccines must be submitted to the NRA of the country where the trials are to be conducted for their review and approval. At the end of the clinical trials, provided the results are satisfactory, an application for marketing authorization can be submitted to the NRA. The most accepted format for the licensing application is the Common Technical Document (CTD), which comprises 5 modules (administrative and legal information; summaries; quality; and nonclinical and clinical information about the product). New products can be registered first in the country of origin and later in other target countries. However, manufacturers may elect to register a new vaccine in user countries if the product is of little interest or applicability in the country of origin. In the case of vaccines against diseases of high public health importance—such as a group A meningococcal (MenA) conjugate vaccine for Africa—and where the vaccine will be purchased and distributed by United Nations (UN) procurement agencies, a prequalification by WHO is also required.

Vaccine prequalification is a WHO-led activity intended to ensure that vaccines purchased by UN procurement agencies will be consistently safe and effective under conditions of use in national immunization programs in low- and middle-income countries (LMICs). Even if the regulatory process has been thorough and effective, WHO must ensure that the product meets the specifications of the UN tender document that have been created to meet the needs of the target population(s) in LMICs [3]. The WHO prequalification program relies on a competent NRA that can provide effective regulatory oversight of the vaccine throughout its lifetime, from the premarket stage to the postmarketing monitoring of quality, safety, and efficacy of the product. Therefore, a precondition for WHO prequalification is licensure by the NRA in the country of origin [3, 4].

The NRA of the country of origin must be “functional” as assessed by an international team of experts using the WHO evaluation criteria [3]. Key requirements include having a legally mandated authority with qualified staff in place, and the capacity for review of submissions of marketing authorization, postmarketing surveillance, and lot release, as well as access to a laboratory for testing vaccines and competent staff for regulatory inspections and regulatory oversight for clinical trials [5–7]. After the prequalification is granted, countries register the vaccines, and the vaccines are then introduced for routine use.

WHO prequalification of vaccines also includes a fast-track option that can be implemented when a vaccine needs to be used as part of an emergency response. Because of the public health importance of MenA meningitis epidemics in Africa, the new MenA vaccine, PsA-TT, was eligible for fast-track prequalification such that the file review, sample testing, and audit of the manufacturing facilities were done in parallel, rather than sequentially. Fast-tracking allows flexibility, and consideration is given to using a streamlined review procedure if the licensing authority in the country of manufacture is eligible or if the licensing authority is partnered with another eligible authority. The streamlined procedure relies upon a detailed review of assessment reports from the NRA with the manufacturer's permission, including inspection reports and the results of tests conducted in the National Control Laboratory of the country of origin to facilitate and accelerate the evaluation process.

At the request of African governments and in response to periodic MenA meningitis epidemics, a new MenA conjugate vaccine was developed through an innovative public–private partnership. The development of the vaccine required regulatory oversight at all development stages and in particular during the African clinical trials, the results of which were used to license the vaccine. WHO in particular was closely involved in the regulatory challenges posed by the clinical trials because the regulatory standards in the meningitis belt countries were limited, as is commonly the case for LMICs. This manuscript outlines the steps taken by WHO and its partners that focused on timely registration and use of this important vaccine while respecting and meeting all international norms.

## THE PSA-TT REGULATORY PATHWAY

Three critical elements ensured timely licensure and registration of PsA-TT:

1. Collaboration between the Drug Controller General of India (DCGI), which is part of the Central Drugs Standard Control Organization (CDSCO), and Health Canada's Centre for Vaccine Evaluation (CVE), which is part of the Biologics and Genetic Therapies Directorate (BGTD), to expedite and enhance the quality of review of the MenA conjugate vaccine;
2. Fast-track review by WHO for prequalification; and
3. WHO assistance for expedited review and licensure of the vaccine in user countries [8].

The collaboration between the DCGI and Health Canada's CVE was part of an ongoing mentoring relationship between Canada's NRA, the BGTD, which is an NRA recognized as functional by WHO and which was asked by WHO to provide training to DCGI to improve its regulatory capacity.

In April 2008, BGTD experts accompanied WHO officials to India to develop a training plan, which was finalized in May 2008. From August 2008 to March 2009, BGTD's CVE provided vaccine regulation training to DCGI staff in workshops held in Canada and India. In addition to the training activities, the DCGI was reorganized, and in April 2009 the DCGI passed the WHO reassessment review. The WHO review also encouraged a continuing collaboration between Health Canada's CVE and the DCGI as well as facilitating that formal review of a new drug application would be a positive experience for both organizations and could further enhance DCGI's vaccine regulatory capacity. The PsA-TT marketing authorization application was chosen as the subject of this joint review, in part because of WHO's urgent need for this particular vaccine. Therefore, Health Canada's CVE supported the DCGI in the review of the regulatory dossier for PsA-TT by conducting its own review of the quality, safety, and efficacy data, in parallel with the DCGI.

## CHRONOLOGY OF EVENTS FOR REGISTRATION AND PREQUALIFICATION OF PSA-TT IN INDIA

### Submission of Dossier in CTD Format

The Marketing Authorization Application for the MenA vaccine (10 µg) was submitted to DCGI by SIIL in a phased manner. The initial submission was in accordance with Drugs and Cosmetics Rules and Schedule Y requirements of the government of India. The submissions were in CTD format specified for registration of pharmaceutical products for human use. The first part of the 13 000-page dossier was submitted in April 2009 and included nonclinical data; a second part was submitted in July 2009 and contained data from phase 1, phase 2, and phase 2/3 clinical studies conducted in India and Africa.

### Review of Dossier and Site Visit by DCGI

The first set of comments by DCGI (modules I, III, and IV of CTD) was issued to SIIL in July 2009. The responses to comments were submitted by SIIL in July, followed by a site audit by DCGI in September 2009.

### Review of Clinical Trial Data and Site Audit Observations by DCGI

The DCGI granted Permission/Notice of Compliance to manufacture MenA conjugate vaccine “for export only” based on the clinical trial data in African populations on 23 December 2009. The issue of a domestic license was withheld by the DCGI pending completion of a review of phase 3 trial data done in India. DCGI's approval of PsA-TT was a major step forward in supporting large-scale introduction of the vaccine in the 26 countries of the African meningitis belt. The approval enabled SIIL to begin producing the vaccine at large scale to meet the projected need of >300 million doses to immunize the 1- to 29-year-old target population in the meningitis belt over the next 10 years. The approval also enabled SIIL to ship 20 million doses of PsA-TT to Africa so that the vaccine was positioned to support initial vaccination campaigns in late 2010.

### Joint On-Site Evaluation by Health Canada's CVE and the DCGI

Health Canada's CVE and the DCGI performed a joint data evaluation and on-site audit at SIIL from 24 to 27 May 2010 to support and accelerate the WHO prequalification of the vaccine.

### WHO Prequalification Review

WHO prequalification review was performed on the basis of review of reports from Health Canada's CVE and DCGI, a review of testing reports by a WHO-contracted laboratory, and an independent audit performed from 8 to 12 March 2010. WHO prequalification was granted on 23 June 2010.

### Submission of Phase 3 Clinical Trial Data of Indian Population to DCGI and Indian Licensure

Phase 3 data of trial conducted in India were submitted to CDSCO on 18 March 2011. PsA-TT licensure for domestic use was granted on 19 December 2011.

## PATHWAY FOLLOWED FOR REGISTRATION IN AFRICAN COUNTRIES

The 3 early introducers (Burkina Faso, Mali, and Niger) were offered the opportunity to review the assessment reports from the prequalification team and those from BGTD and CDSCO in Geneva. Other countries were invited to follow the expedited review procedure proposed and published by WHO [8].

## RESULTS AND DISCUSSION

The licensure of PsA-TT was undertaken by the DCGI based on the application filed by SIIL, recognizing that WHO was interested in providing a safe, effective, and affordable MenA conjugate vaccine to African countries in the meningitis belt. Because meningococcal disease is not prevalent in India, the initial licensure activity focused on making the vaccine available to Africa. For the purpose of PsA-TT licensure, the DCGI also accepted parallel review of the vaccine with Health Canada's CVE, which served as a second reviewing NRA. This step was critically important to ensure that a streamlined approach that included Health Canada's CVE was in place to evaluate the PsA-TT dossier.

As per the Indian regulations, a marketing authorization for domestic use of any vaccine requires the performance of clinical trial(s) in India [9]. Hence, the first approval granted for the PsA-TT vaccine was for export purposes only. The review of the CTD by DCGI was performed on a rolling basis with submission of modules I, III, and IV in April 2009 and that of modules II and V in July 2009. The marketing authorization (for export only) was granted 6 months later, in December 2009.

WHO then based its prequalification on the dossier assessment reports done by the DCGI and Health Canada instead of performing an independent evaluation of the dossier, testing of samples, and site audit of the manufacturing facilities. WHO conducted an independent audit of the facilities and granted the prequalification in June 2010, 6 months after approval by DCGI.

The most challenging step was achieving timely registration in the user countries. Burkina Faso, Mali, and Niger were targeted for early introduction of the vaccine, and WHO invited NRA staff from these countries to follow an expedited review in Geneva that was based on the assessment reports from DCGI, Health Canada, and WHO. This process facilitated the registration of the vaccine in Burkina Faso and Niger, where the process took 3 and 8 months, respectively. However, the approach was not successful in Mali where, for unclear reasons, the registration process took 16 months.

After the initial 3-country introduction, the PsA-TT rollout was scheduled in a stepwise manner for the rest of the 23 countries of the meningitis belt. The rollout schedule necessitated timely vaccine registration in these countries. WHO proposed that countries follow an expedited registration procedure given that PsA-TT was WHO prequalified. The need for rapid introduction of PsA-TT was considered an excellent opportunity to advocate for the use of an accelerated registration procedure in meningitis belt countries. The WHO-expedited procedure was originally written to prioritize countries that procured vaccines from the United Nations Children's Fund (UNICEF) but lacked the expertise to review a biological product and, in particular, vaccines. The second priority would be countries that procured some vaccines from UNICEF but also self-procured other vaccines and had expertise to review a vaccine dossier but had not yet registered a new vaccine for their national immunization programs. These NRAs would require the investment of significant training resources. Last, there were countries that procured vaccines directly and had the required expertise to review a vaccine dossier but, for efficiency and resource-saving reasons, elected to apply the expedited review procedure for some vaccines and to reserve the right to perform the full review for other vaccines. Such countries may decide, for example, to apply the expedited review procedure for traditional vaccines with a long history of use globally while they apply a longer full-review procedure for novel vaccines recently introduced in the market.

Two workshops on the use of the expedited review procedure were held, one for English-speaking and one for French-speaking countries, to enable them to use the procedure for registration of PsA-TT. The 5-day workshops included the review of documents on the production and control of PsA-TT; inspection of packaging, inserts, and samples of the vaccine; and a mock review of a vaccine and the preparation of a registration report. Facilitators from WHO and Health Canada took participants through the epidemiology of meningitis, basic vaccinology, development of PsA-TT, the manufacturing process, and testing performed, as well as how to review dossiers and to prepare an evaluation report.

Representatives from the NRAs requested WHO to share the report of the assessment that was the basis for the prequalification. At the end of the workshops, participants were expected to return to their institutions, receive and review dossiers and samples submitted by the manufacturer, and make a decision on registration of the product within 30 days of receipt of the submission and the reports from WHO.

The workshops were very successful and feedback from participants was positive, indicating that they had learned about the product, the manufacturing process and quality control, how to critically review the documentation against the WHO recommendations, and how to check the samples and review the labels and package inserts.

Table 1 shows the dates when the marketing authorization applications were submitted by SIIL and when the marketing authorizations were granted in the countries of the meningitis belt, and includes the regulatory pathway that countries followed. In spite of the priority given to the marketing authorization and prequalification of PsA-TT, the marketing authorization in user countries was not easy to obtain. SIIL submitted a marketing authorization application to 28 countries in Africa. Fourteen countries reported having followed the national registration procedure according to their own regulations, with the process taking between 6 months (Rwanda) and 34 months (Kenya). Two of the 3 early-introduction countries, Niger and Burkina Faso, followed the national procedure but used the support provided by WHO, resulting in registration in 3 and 8 months, respectively. In contrast, the Mali registration took 16 months despite the support provided by WHO in the review process. Four countries reported following the expedited review procedure recommended by WHO, and the registration process took 15–25 months.

**Table 1.** Registrations of PsA-TT (MenAfriVac) in African Countries of the Meningitis Belt

No	Country	Status of Registration	Date of Submission	Date of Registration	Registration Procedure
1	Niger	Registered	Jan 2010	Aug 2010	National registration procedure
2	Burkina Faso	Registered	Jul 2010	Oct 2010	National registration procedure
3	Ghana	Registered	May 2010	Jan 2011	National registration procedure
4	Guinea	Registered	Aug 2010	May 2011	National registration procedure
5	Mali	Registered	Feb 2010	May 2011	National registration procedure
6	Uganda	Registered	Aug 2010	Jan 2012	National registration procedure
7	Sudan	Registered	Sept 2011	Feb 2012	National registration procedure
8	Rwanda	Registered	Nov 2011	Apr 2012	National registration procedure
9	Senegal	Registered	May 2011	July 2012	National registration procedure
10	Chad	Registered	Apr 2010	Feb 2012	National registration procedure
11	Kenya	Registered	May 2010	March 2013	National registration procedure
12	Nigeria	Registered	Sept 2010	May 2013	National registration procedure
13	Côte d'Ivoire	Registered	Jul 2010	Dec 2011	WHO-expedited procedure
14	Benin	Registered	Jul 2010	Aug 2012	WHO-expedited procedure
15	Tanzania	Registered	Dec 2010	March 2012	WHO-expedited procedure
16	Cameroon	Registered	Apr 2010	May 2012	National registration procedure
17	The Gambia	Registered	Nov 2011	Jul 2013	WHO-expedited process
18	Mauritania	Registered	Nov 2011	Jul 2014	National registration procedure
19	Togo	Special import authorization	Nov 2011	Sep 2014	Exceptional authorization for vaccine import with 1-time validity
20	Ethiopia	Registered	Apr 2012	Sep 2014	To be confirmed

Countries that are not yet registered, in which introductions are expected over the next couple of years, include Burundi, Central African Republic, Democratic Republic of Congo, Eritrea, Guinea-Bissau, and South Sudan.

Abbreviation: WHO, World Health Organization.

The status of registration remains pending in 6 countries: Burundi, Central African Republic, Democratic Republic of Congo, Eritrea, Guinea Bissau, and South Sudan. The actual pathway followed for registration in these countries remains unclear.

The timelines taken for registration in the African countries of the meningitis belt was unjustifiably long in the majority of cases. These delays, however, did not hamper the use of the vaccine, which was introduced in the target countries according to the predefined schedule. This means that either the countries gave some sort of waiver of the registration to allow the use of the vaccine pending completion of the registration process, or they decided to introduce the vaccine independently (ie, as an unregistered product).

The collaboration established between the Indian and Canadian NRAs and WHO facilitated the registration in India as well as the prequalification by WHO. However, the registration process in user countries did not move as quickly as hoped. One important impediment was that some countries already had in place national regulations that stipulated a full evaluation of the product, including in some cases requirement for a CTD dossier, testing of samples, site inspection, and/or specific labeling requirements. Although the registration procedure that was published and advocated by WHO was not embedded in country regulations, the support provided by WHO was successful in facilitating the scientific review of the dossier. In short, the proposed WHO registration procedure did not alter the additional requirements that certain countries had to follow. Another important factor that may have affected registration timelines can be related to the administrative steps preceding or following the technical review of the product such as the format of the submission, requirements for an in-country SIIL agent, and internal approval of the assessment report by designated committees to grant the marketing authorization.

## CONCLUSIONS

The licensure and rapid introduction of PsA-TT has proven to be a major success and serves as an excellent example of the power of committed partnerships to overcome political, operational, and regulatory hurdles to make this vaccine available in meningitis belt countries in a timely manner. However, and in spite of WHO support, there were unexpected delays in national registration procedures for some countries, which suggests that more work needs to be done to further improve country regulatory processes. The WHO-facilitated procedure used for licensure in India and to accelerate prequalification of the vaccine worked well to prequalify PsA-TT in a timely manner. The PsA-TT case study also made it possible to identify gaps in the WHO-expedited review procedure for registration of the vaccine in user countries and to address them. WHO has now revised the procedure and seeks to establish collaborative agreements with countries where NRAs are willing to adopt abbreviated pathways for registration of priority vaccines [10].
